# Developing the EPA guidance of pharmacological treatment of schizophrenia – results of a Delphi process

**DOI:** 10.1192/j.eurpsy.2024.1794

**Published:** 2025-01-30

**Authors:** Peter Falkai, Elias Wagner, Miriam John, Vladislav Yakimov, Silvana Galderisi, Istvan Bitter, Geert Dom, Andrea Schmitt, Wolfgang Gaebel, Bernardo Carpiniello, Alkomiet Hasan

**Affiliations:** 1 Department of Psychiatry and Psychotherapy, LMU University Hospital, Ludwig-Maximilians-University of Munich, Munich, Germany; 2 DZPG (German Center for Mental Health), Partner Site München/Augsburg, LMU Munich, Germany; 3 Department of Psychiatry, Psychotherapy and Psychosomatics, Faculty of Medicine, University of Augsburg, Augsburg, Germany; 4 Evidence-Based Psychiatry and Psychotherapy, Faculty of Medicine, University of Augsburg, Augsburg, German; 5 International Max Planck Research School for Translational Psychiatry (IMPRS-TP), Munich, Germany; 6 Department of Mental and Physical Health and Preventive Medicine, University of Campania Luigi Vanvitelli, Naples, Italy; 7 Department of Psychiatry and Psychotherapy, Semmelweis University, Budapest, Hungary; 8 Collaborative Antwerp Psychiatric Research Institute, Wilrijk, Belgium; 9 Department of Psychiatry and Psychotherapy, Heinrich Heine University, Düsseldorf, Germany; 10 Section of Psychiatry, Department of Medical Sciences and Public Health, University of Cagliari, Cagliari, Italy; 11 DZPG (German Center for Mental Health), Partner Site München/Augsburg, Augsburg, Germany

**Keywords:** Schizophrenia, Guideline, Europe, Treatment, Antipsychotics

## Abstract

**Background:**

The development of guidelines is time-consuming and cost-intensive. The heterogeneity of clinical practice, evidence, and patients’ needs is an issue across Europe. An European core guidance for a specific psychiatric disorder may help to overcome this issue. Here, we present a progress report on the European Psychiatric Association (EPA) proof-of-concept approach to develop a European consensus guidance on the pharmacological treatment of schizophrenia.

**Methods:**

All national psychiatric associations in Europe were contacted to provide their schizophrenia guidelines. Six guidelines were rated by three experts, experienced in the development of national and international guidelines, from three different countries (Italy, Hungary, and Germany), and the German schizophrenia guideline published in 2019 was found to have the highest quality. For this proof-of-concept approach, 45 recommendations on the pharmacological treatment of schizophrenia from the German guideline were evaluated in a two-step Delphi process to determine their acceptability throughout the European continent.

**Results:**

44 experts participated in the first round and 40 experts in the second round of the Delphi process. Agreement among the involved experts was reached for 75% of the presented recommendations from the German schizophrenia guidelines. 11 out of 45 recommendations (24.4%) did not reach this level of agreement.

**Conclusions:**

This progress report highlights the possibility of developing a pan-European core guidance on the pharmacological treatment of schizophrenia by adapting national guidelines and reconciling their recommendations. However, several barriers in this adaptation process, such as non-agreement in recommendations with strong scientific evidence in the reconciling process, were identified and must be considered when developing the final guidance.

## Introduction

Medical guidelines are systematically developed tools to assist physicians, psychologists, and other health-care professionals as well as patients and relatives in the decision-making process of a given treatment. Thus, guidelines promote the transparency of medical decisions. In that regard, guidelines evaluate and summarize the scientific evidence, help to determine the right and individual treatment for a given patient by weighting risk–benefit ratios, and are considered to improve the quality of medical treatments [1]. However, the development of guidelines is complex, cost-intensive, and needs substantial knowledge of the concept of evidence-based medicine [2, 3].

There is a substantial heterogeneity in clinical practice across European countries, which is mirrored in differences in treatment guidelines [4]. To harmonize guideline recommendations across Europe and to optimize the resources used by national approaches, the European Psychiatric Association (EPA) aims to develop a European core guidance on the pharmacological treatment of schizophrenia. If successful, this process should be extended to other treatments such as psychotherapy or psychosocial treatments and other disorders. The report is presently in a progressive state, currently based on the German evidence- and consensus-based schizophrenia guidelines. The aim of this report is to eventually create an overall European guidance for schizophrenia. This European guidance shall be adapted to European country-specific requirements and conditions by considering each county’s guideline competences intimately involving National Psychiatric Associations (NPAs).

Currently, we have 19 national treatment guidelines on schizophrenia available from 44 NPAs of the EPA. Worldwide, there are many more published with differing quality and scope (see a brief overview elsewhere: [5–7]). Every guideline has its own emphasis, target group, evidence-evaluation strategy, and presentation, but most guidelines overlap in a significant number of recommendations. This applies in particular to aspects of antipsychotic treatment. Thus, this overlap may lay the foundation for a European core guideline for an evidence-based, standardized, ethical, and cost-effective treatment of schizophrenia throughout Europe, targeting patients’ benefits. In that regard, EPA decided as a very first step to create a “Guidance paper on the pharmacological treatment of Schizophrenia” to build a consensus on how to best treat this disorder pharmacologically within their member associations. If successful, this concept could be the basis for future development of EPA core guidance publications for major mental disorders allowing an up-to-date knowledge transfer from published science into routine clinical care. This harmonized process can then be followed by further development of these core guidance documents to European or national living guidelines. Living guidelines allow for a fast update of recommendations as soon as new and relevant research becomes available [8] reducing the gap between publications and recommendations. As detailed below, we were able to identify the German evidence-and consensus-based guideline [5] as the guideline with the highest scientific quality within EPA. This guideline was used as a starting point for the development, coordination, and discussion of the planned core guideline. In this process, the NPAs of the EPA, the Global Alliance of Mental Illness Advocacy Network (GAMIAN) Europe, and the European Federation of Associations of Families of People with Mental Illness (EUFAMI) have been involved. Here, we report on the progress of this development.

## Methodology

All 44 NPAs of the EPA were invited to make their respective national schizophrenia guidelines available, mounting up to 19, which were collected via email by the EPA head office. Three reminders were sent out. Reasons for the gap between 19 guidelines and 44 NPAs were, for example,the lack of availability of clearly described national guidelines or non-responses of the respective NPA. Out of those 19 guidelines, eight guidelines would have been potentially eligible as they were published no more than 5 years ago (one further could not be translated during the project period), and included pharmacological and non-pharmacological treatments of schizophrenia. The EPA president (PF) selected three schizophrenia experts (SG, IB, AH) based on their experience in developing guidelines from three different countries (Italy, Hungary, and Germany). They independently rated the methodological quality out of six of these national schizophrenia guidelines stemming from Germany, Ukraine, Finland, the UK, Slovakia, and Switzerland using the AGREE-II tool [9]. The guidelines from Norway and Croatia arrived too late to be involved in this process. Thus, only six guidelines were evaluated by the experts. Based on the AGREE-II tool, the minimum value was 1 (strongly disagree) and the maximum value was 7 (strongly agree). The schizophrenia guideline of the German Association for Psychiatry, Psychotherapy and Psychosomatics (DGPPN) [5] received the highest mean final evaluation score of 6.00 ± 1.00 points and was therefore selected to be the basis for the subsequent Delphi process. The guidelines of Ukraine (2.67 ± 0.58), Finland (3.33 ± 1.53), UK (5.00 ± 1.00), Slovakia (3.67 ± 0.58), and Switzerland (4.67 ± 1.56) reached lower rankings. For the Delphi process, a consensus group was developed consisting of schizophrenia experts of which 44 were selected from 26 NPA presidents (no more than 2 from one country) and five were nominated both from EUFAMI and GAMIAN-Europe. In the first and second round of the online Delphi process, which took place between January and April 2023, the 45 recommendations (including two statements) on pharmacotherapy or biological treatment (except catatonia and comorbidities such a sleep-disturbances or agitation) from the schizophrenia guideline of the German Association DGPPN were rated (agree vs. not agree with the recommendation). The threshold criterion for a consensus recommendation was ≥75% of agreement in the second round, which matches recommendations of the literature ranging between 70 and 80% [10]. Ethical approval for this project was obtained prior to study start from the Medical Faculty, LMU University Hospital, Munich, Germany (reg. nr. 22-0887 KB).

## Results

In total, 68 experts were named by the respective NPAs out of 32 countries plus respectively two from GAMIAN and EUFAMI. In the end, 44 experts (45.5% female) participated in the first round of the Delphi survey, with a mean age of 53.16 ± 8.77 years and a mean professional experience with people with schizophrenia of 25.64 ± 10.03 years. 40 participated in the second round of the Delphi survey. Please see Table 1 for more demographic information of the sample. 34 out of 45 recommendations (75.6%) reached a level of agreement above 75% showing a good consensus across Europe on how to offer evidence-based pharmacological treatments to people with schizophrenia. This was based on scientific evidence and a rating scale between “agree,” “disagree” or “agree with changes.” 11 out of 45 recommendations (24.4%) did not reach this level of agreement. Table 2 highlights the detailed results of the final Delphi process. Though not reaching the 75% level of agreement, most of those 11 recommendations had still a substantially higher frequency of agreements compared to non-agreement. Remarkably, seven recommendations (64%) with no agreement were based on meta-analyses or randomized-controlled trials, meaning that no consensus was reached despite a high-level scientific evidence, as they did not seem to meet the clinical experience in the given country. Moreover, two of these recommendations (18%) had the highest strength of recommendation (A) in the source guideline [3, 9]. Please see Table 2 for a comprehensive description of all recommendations and the voting results of the second Delphi round.

**Table 1.** Participating NPAs and other associations and their representatives. *N* = sample size; SD = standard deviation

Variables	N	Mean ± SD
Age (years)	43	53.16 **±** 8.77
Years of professional experience with people with schizophrenia	44	25.64 **±** 10.03
	**N**	**Frequency (%)**
Gender (m/f)	24/20	54.5/ 45.5
Participating Associations (*N* = 26 with 44 experts)	44	100
Austrian Society for Psychiatry and Psychotherapy	1	2.3
Belarusian Psychiatric Association	1	2.3
Belgium – Flemish Association of Psychiatry	2	4.5
Croatian Psychiatric Association	1	2.3
Czech Psychiatric Association	2	4.5
Finnish Psychiatric Association	2	4.5
French Congress of Psychiatry	2	4.5
Society of Georgian Psychiatrists	2	4.5
German Association for Psychiatry, Psychotherapy and Psychosomatics (DGPPN)	1	2.3
Hungarian Psychiatric Association	2	4.5
The College of Psychiatrists of Ireland	2	4.5
Israel Psychiatric Association	1	2.3
Italian Psychiatric Association	1	2.3
Lithuanian Psychiatric Association	2	4.5
Society of Psychiatrists, Narcologists, Psychotherapists and Clinical Psychologists from Republic of Moldova	2	4.5
Norwegian Psychiatric Association	2	4.5
Polish Psychiatric Association	1	2.3
Romanian Association of Psychiatry and Psychotherapy	1	2.3
Serbian Psychiatric Association	2	4.5
Slovak Psychiatric Association	2	4.5
Spanish Society of Psychiatry	2	4.5
Swiss Society for Psychiatry and Psychotherapy	2	4.5
Psychiatric Association of Turkey	2	4.5
Royal College of Psychiatrists	2	4.5
Member of GAMIAN Europe	2	4.5
Not specified	2	4.5

**Table 2.** Recommendation survey results (Recommendations that have not reached the 75% agreement are highlighted in bold)

Recommendation	Contents of recommendations (cited according to [1, 2]) (strength of recommendation)	Total *N*	Agree (*n* / %)	Disagree (*n* / %)	No response (*n* / %)
15	We recommend embedding pharmacotherapy in a holistic treatment concept that includes general and specific psychotherapeutic and psychosocial measures and psychiatric treatment, depending on the differential indication (GCP).	40	40/100%	0	0
16	We recommend telling the patient at the start of pharmacotherapy about the acute and long-term effects and adverse effects of the drugs (risk–benefit evaluation) and actively involving patients in the decision-making process (shared decision making, see Module 3). We also recommend presenting the advantages and disadvantages of the treatment and possible alternatives in clear language and explaining technical terms (GCP).	40	39/97.5%	0	1/2.5%
17	Before starting pharmacotherapy, we recommend performing laboratory tests, as shown in Table 9 [of the DGPPN guideline] and recording an ECG. We recommend ruling out pregnancy in women of child-bearing age (GCP).	40	32/80%	3/7.5%	5/12.5%
18	We recommend that the decision about the suitable antipsychotic and route of administration is made jointly by the service user and treating doctor. We recommend considering and discussing the following: The clinical syndrome to be treated Previous experience of effects and side effects of one or more drugs during treatment to date Advantages and disadvantages of the respective drug Metabolic, motor, cardiovascular or hormonal/sexual side effects (see Table 9 [of the DGPPN guideline]) Benefits and risks of forgoing treatment with antipsychotics The service user’s preferences Sex-specific aspects, patient’s age, and comorbidities We recommend taking into consideration any treatment agreements or crisis plans that the patient may have (see also Module 4c [of the DGPPN guideline]). We recommend continually reviewing the risk–benefit assessment in the course of treatment and taking appropriate measures if there are any changes (GCP).	40	38/95%	0	2/5%
**19**	**There is insufficient evidence of any differences in the efficacy of oral, intramuscular, and intravenous antipsychotics in the treatment of the acute illness. We recommend using parenteral administration only in very exceptional cases. We recommend choosing the oral route of administration in cooperative patients, unless the patient requests a different route, because it is the least invasive, has similarly good efficacy and best ensures patient autonomy (GCP).**	**40**	**27/67.5%**	**11/27.5%**	**2/5%**
20	Therapeutic drug monitoring (TDM) may be considered in case of adverse drug reactions, clinical non-response, suspected drug interactions and suspected noncompliance. We recommend basing the use and frequency of TDM on the 2017 update of the Arbeitsgemeinschaft für Neuropsychopharmakologie und Pharmakopsychiatrie (AGNP) guidelines (GCP).	40	34/85%	2/5%	4/10%
21	In case of treatment resistance, we suggest reaching a serum level of clozapine of at least 350 ng/ml, as long as there are no tolerability issues (B).	40	30/75%	2/5%	8/20%
22	We recommend offering antipsychotics at a dose that is within the range recommended by the respective international consensuses and is as low as possible and as high as necessary (lowest possible dose). Particularly in first episodes of the illness, we recommend choosing the dose in the lower range because people with a first episode have a higher sensitivity for side effects and an overall better response to a lower dose (A).	40	36/90%	0	4/10%
23	We suggest offering continuous antipsychotic pharmacotherapy for relapse prevention (B).	40	33/82.5%	3/7.5%	4/10%
24	If the patient is stable and there are reasons why continuous long-term medication cannot be continued (e.g. lack of acceptance), we suggest offering stepwise dose reduction, followed by supervised intermittent treatment combined with targeted early intervention in case of prodromal symptoms of an impending relapse (GCP).	40	30/75%	4/10%	6/15%
25	After a decision has been made that the dose of antipsychotics can be reduced, we suggest offering a dose reduction, taking into account the recommended treatment duration (Recommendations 36 and 37). We suggest reducing the dose in very small steps at intervals of 6 to 12 weeks, depending on the patient’s preferences. Furthermore, we suggest involving the patient’s family and close confidants and taking into consideration the overall treatment plan, course of treatment to date and tolerability of the existing antipsychotic medication (GCP).	40	36/90%	0	4/10%
26	A reduction and possible discontinuation of antipsychotics at any stage of the illness in terms of shared decision-making between the patient and the treating doctor may be considered, as long as sufficient stability and psychosocial support and regular, ongoing monitoring of symptoms are guaranteed and there are no indications that the patient is a danger to self or others. We recommend informing every patient about the increased risk of relapse after discontinuation. Suggestions for dose reduction and discontinuation can be found in the background text (GCP).	40	35/87.5%	1/2.5%	4/10%
27	We suggest that after discontinuing antipsychotics, signs and symptoms of a relapse should be continually monitored for at least two years as part of the overall treatment plan (GCP).	40	36/90%	0	4/10%
28	We recommend that in cases of insufficient response to treatment despite adequate treatment duration, practitioners reassess the diagnosis, psychiatric and medical comorbidities, adherence, illegal substance use, presence of debilitating side effects, effective dosing (incl. serum level monitoring and confirmation of the indication), environmental factors (e.g. stress, high expressed emotions) and effective treatment duration. We recommend evaluating these secondary causes for insufficient treatment and, if necessary, addressing them before offering to change the medication (GCP).	40	36/90%	0	4/10%
**29**	**We recommend evaluating the response status after two weeks (at the latest after four weeks) by using a suitable scale (ideal: PANSS, BPRS; easier: CGI) (A). In case of lack of response (CGI unchanged or worse [CGI < 3]) despite adequate dosing and after excluding secondary causes, we recommend offering the patient a switch to an antipsychotic with a different receptor binding profile, with the aim to achieve response (GCP).**	**40**	**27/67.5%**	**4/10%**	**9/22.5%**
30	If response is adequate but there are tolerability issues, an early switch to a drug with a different side-effect profile may be considered (GCP).	40	33/82.5%	0	7/17.5%
31	Every change in medication can result in a worsening of symptoms or an increase in side effects. When switching to a different antipsychotic, the cross-taper or overlap-and-taper strategy may be considered. The stop-start strategy may be considered if the antipsychotic has to be discontinued immediately because of side effects. We suggest considering equivalence doses when changing antipsychotic treatment (GCP).	40	36/90%	0	4/10%
32	We recommend offering pharmacological treatment with an antipsychotic as a monotherapy with the goal to reduce psychotic symptoms (A).	40	35/87.5%	0	5/12.5%
33	During the acute phase, we recommend reviewing and documenting the psychopathological findings at appropriate intervals so that a danger to self and others can be recognized in a timely manner and treatment response can be evaluated (GCP).	40	36/90%	0	4/10%
34	In first-episode schizophrenia, we recommend offering antipsychotics to reduce psychotic symptoms, after considering the respective risk–benefit. The risks of the treatment can be derived from the respective side-effect profile of the antipsychotics used. Because there are few differences in the efficacy of the various drugs and the response rate is high in first-episode schizophrenia, we recommend basing the choice of antipsychotic primarily on the side-effect profile (A).	40	34/85%	2/5%	4/10%
**35**	**In first-episode schizophrenia, we suggest offering antipsychotic treatment as early as possible. Depending on the psychopathology, treatment setting and patient preferences, in first-episode schizophrenia practitioners may consider waiting a few days to weeks before initiating antipsychotic pharmacotherapy as part of a psychosocial overall plan, while closely monitoring the psychopathology (GCP).**	**40**	**25/62.5%**	**8/20%**	**7/17.5%**
36	After an individual risk–benefit evaluation has been performed, we recommend offering people with schizophrenia (first episode and multiple episode) antipsychotic treatment for relapse prevention (A).	40	34/85%	0	6/15%
37	For relapse prevention, we recommend offering the antipsychotic that has already resulted in good treatment response or remission, as long as no tolerability issues exist (A). When choosing the antipsychotic for relapse prevention, we recommend considering the service user’s preferences and previous experiences, as well as the differing risks of side effects such as tardive dyskinesia, sedation and cardiac, metabolic, endocrine and other effects (GCP).	40	34/85%	1/2.5%	5/12.5%
38	Like oral antipsychotics, depot antipsychotics are effective for relapse prevention and show no relevant differences in efficacy. Because of their guaranteed administration and good bioavailability, depot antipsychotics are an effective alternative to oral medication, and we suggest offering depot antipsychotics as an alternative treatment for relapse prevention (B).	40	33/82.5%	2/5%	5/12.5%
39	Because there is insufficient evidence for superior efficacy of any individual depot antipsychotic, we suggest choosing a depot antipsychotic on the basis of the side-effect profile and the desired injection interval. Before starting treatment with the depot form of an antipsychotic, we suggest ensuring its efficacy and tolerability by offering the oral form of the respective antipsychotic for at least several weeks (GCP).	40	32/80%	4/10%	4/10%
**40**	**In case of predominant negative symptoms, we suggest offering amisulpride (at a low dose) or olanzapine. We suggest avoiding the use of strong D2 receptor blockers by using antipsychotics with a suitable profile or avoiding high-dose treatments (B).**	**40**	**12/30%**	**18/45%**	**10/25%**
**41**	**In case of inadequate response to antipsychotic monotherapy, we suggest offering additional treatment with antidepressants to people with schizophrenia and predominant negative symptoms (B).**	**40**	**21/52.5%**	**8/20%**	**11/27.5%**
42	Before diagnosing drug treatment resistance, we recommend excluding pseudoresistance. We recommend considering the following characteristics: adherence, illegal substance use, the presence of debilitating side effects, comorbidities (e.g. trauma), effective dosing (incl. easuring serum levels and checking for interactions), and environmental factors (e.g. stress, high expressed emotions) (GCP).	40	33/82.5%	0	7/17.5%
43	In cases of proven antipsychotic treatment resistance and after evaluating the risk–benefit profile and providing information, and in accordance with the necessary accompanying tests, we recommend offering an attempt to treat the existing psychotic symptoms with clozapine (A).	40	33/82.5%	0	7/17.5%
**44**	**If clozapine is not tolerated, treatment with olanzapine or risperidone may be suggested (GCP).**	**40**	**12/30%**	**14/35%**	**14/35%**
45	If there is no treatment response, we suggest *not to* increase antipsychotic doses above the approved range (B).	40	29/72.5%	5/12.5%	6/15%
46	In case of drug treatment resistance, we recommend first offering treatment with an antipsychotic in monotherapy (A). A combination of two antipsychotics may be suggested, with monitoring of side effects and interactions, if adequate response is not achieved with monotherapy with three different antipsychotics, including clozapine (GCP). We recommend documenting this approach and, if there is still no treatment response, discontinuing this strategy (GCP).	40	31/77.5%	3/7.5%	6/15%
**47**	**In case of drug treatment resistance, we recommend *not to* offer augmentation treatment with carbamazepine, lithium, lamotrigine, or valproate as a standard treatment to improve general, positive, or negative symptoms or aggression (A).**	**40**	**20/50%**	**7/17.5%**	**13/32.5%**
**48**	**In case of clear antipsychotic treatment resistance after adequate treatment at a high enough dose for a long enough time, we suggest offering ECT as an augmentation treatment with the aim to improve the overall clinical condition (B).**	**40**	**28/70%**	**3/7.5%**	**9/22.5%**
**49**	**In case of antipsychotic treatment resistance, we suggest offering treatment with low-frequency rTMS at 1 Hz, applied over the left temporal lobe, as part of an overall treatment plan in people with schizophrenia and persistent acoustic hallucinations (B).**	**40**	**18/45%**	**8/20%**	**14/35%**
**50**	**In case of drug treatment resistance, people with schizophrenia and persistent negative symptoms may be offered treatment with high-frequency rTMS at 10/20 Hz, applied over the left dorsolateral prefrontal cortex, as part of an overall treatment plan (0).**	**40**	**15/37.5%**	**10/25%**	**15/37.5%**
51	In case of severe agitation, anxiety and inner restlessness, add-on treatment with benzodiazepines (e.g. lorazepam) may be considered for a limited period of time and in accordance with the applicable recommendations (GCP).	40	31/77.5%	0	9/22.5%
52	We recommend not only informing people with schizophrenia, family members, and close confidants about possible adverse drug reactions but also advising them about the associated symptoms and respective treatment options (GCP).	40	33/82.5%	0	7/17.5%
53	We recommend actively enquiring about and documenting antipsychotic-induced adverse drug reactions and, if suspected, offering suitable tests and treatment (GCP).	40	33/82.5%	0	7/17.5%
54	Depending on the severity of the antipsychotic-induced adverse drug reactions, after a risk–benefit evaluation we recommend offering a dose reduction, switch to a different drug or discontinuation (GCP).	40	33/82.5%	0	7/17.5%
55	At the start of antipsychotic treatment or at the latest after the occurrence of strong, antipsychotic-induced weight gain (>7% of baseline weight), we recommend offering psychotherapeutic and psychosocial interventions (nutrition advice, psychoeducation, exercise programmes) to prevent weight gain or to reduce weight (A).	40	33/82.5%	0	7/17.5%
**56**	**If there is strong weight gain and it is necessary to continue the current antipsychotic medication, after performing the specified psychotherapeutic and psychosocial interventions (see Recommendation 55 and background text [of the DGPPN guideline]) we recommend offering treatment with metformin (first choice) or topiramate (second choice) for weight reduction, taking into account the risks of an additional drug treatment (A).**	**40**	**25/62.5%**	**4/10%**	**11/27.5%**
57	We recommend informing service users, family members and close confidants, as well as carers, about the necessary monitoring tests* (see Table 9 [of the DGPPN guideline]), and we recommend implementing the monitoring tests as part of the overall treatment plan (GCP). *The legal regulations regarding confidentiality must hereby be observed.	40	32/80%	0	8/20%
Statement 2	We recommend informing people with a relapsing illness course, their family members, and close confidants that the relapse risk doubles one year after discontinuing antipsychotic treatment (27% if treatment is continued, 65% if it is discontinued) and remains higher for the next 3–6 years (22% if treatment is continued, 63% if it is discontinued).	40	35/87.5%	1/2.5%	4/10%
Statement 3	The duration of treatment is influenced by a number of variables and individual factors, such as the severity of the index episode, treatment response, adverse drug reactions, motivation of the service user, family history, illness severity, the psychosocial situation, the available psychotherapeutic and psychosocial treatment options and the overall health care situation, which should be considered in each individual situation.	40	34/85%	2/5%	4/10%

## Discussion

Here, we present a progress report on developing an EPA core guidance for the treatment of schizophrenia based on national guidelines. This first step should lay the foundation for further guidance publications and help to currently develop state-of-the-art tools to guide clinicians, patients, and other stakeholders in times of scarce time and financial resources. Our proof-of-concept approach focused on the pharmacological treatment of schizophrenia but will be extended to psychotherapeutic and psychosocial treatments. We were able to show the feasibility of this approach and the agreement on 75% of all recommendations on the pharmacological treatment from the German schizophrenia guideline [5, 11] showing that it is possible to scale a national guideline to other countries. However, prior to the final adoption of European core guidance, a discussion panel in addition to the Delphi processes used here is needed. This can be explained by the fact that our experts did not agree on several evidence-based recommendations that have been rooted in strong scientific evidence. This must be especially questioned for recommendations with an A-level recommendation, such as using metformin to prevent weight gain and not-to-use mood stabilizers to augment antipsychotic treatment. One should be aware that for metformin not only meta-analyses highlight possible advantages of this approach [12, 13], but that also one guideline based on the GRADE approach supports this strategy [14]. At this stage, we may speculate whether the uncertainty of evidence or uncertainties [12] in the application has resulted in the here reported discrepancies. Neurostimulation using electroconvulsive therapy (ECT) or repetitive transcranial magnetic stimulation (rTMS) did also result in non-agreement. One could speculate whether the inconclusive data regarding rTMS, the non-availability in some countries, or the general scepticism regarding ECT (e.g. due to lack of information) may explain these findings. A relevant limitation of the Delphi process stems from the fact that some recommendations on pharmacological treatment from the German schizophrenia guideline combine multiple statements. Thus, an expert might agree with one but disagree with another statement and this information is not adequately captured by the rating process. This aspect must be taken into consideration during the development of the EPA core guidance for the treatment of schizophrenia. Interestingly, 18% (2/11) of the recommendations with less than 75% agreement pertain to the treatment of negative symptoms, perhaps reflecting the currently limited options of available pharmacological treatments for this domain of schizophrenia psychopathology [15]. In the used German guidelines, especially CBT and training of social skills received high recommendation levels [5, 11]. It is important to note that during the country-specific approval process of the German guideline, all recommendations received >75% agreement. Importantly, to develop a European core guidance, we must ensure that during the nominal group process no personal opinions, conflicts of interest or special interests influence the voting results.

However, we were able to show the feasibility of such an approach. This progress report will guide the next steps including developing a full set of EPA recommendations for the treatment of schizophrenia and upon finalization and acceptance by the NPAs other guidelines may be further developed in a related manner. Thus, we plan to implement an up-to-date guidance paper in terms of overall European guidance for the treatment of schizophrenia. We plan to adapt the guidance paper to European country-specific requirements and conditions, considering each country’s guideline competences in terms of feasibility and applicability. To reach this goal, each recommendation could be reviewed by two authors who can make recommendations for updates of the text and of the supporting references as well as of the strength of evidence with a good approval process prior to submission. Moreover, the NPA boards should also have the opportunity to review and approve the planned guidance. Changes will then be discussed, revised, and approved by all authors, and presented during an online meeting of the authors. This new guidance paper will be developed in such a way that it can be transferred to a living guideline. Living guidelines have experienced an upswing during the COVID pandemic. They are an optimization of the established guideline development process by adding the option that individual recommendations can be updated as soon as relevant new evidence is available [8]. Concepts of how to develop living guidelines on a national level are available (e.g. [16]) and to take the next steps on a European level, such manuals describing the process of developing a living guideline must be adapted as well. In general, we are aware that guideline and guidance implementation remains in many cases insufficient [17–20]. Several barriers including personal factors (e.g. lack of motivation, lack of awareness, lack of knowledge), guideline-related factors (e.g. guidelines are outdated), external factors (e.g. difficulties in accessing guideline), or lack of resources (e.g. no possibility to implement a treatment due to the financial situation in the given healthcare area) have been identified in implementing guidelines [19]. This must be kept in mind when developing a pan-European EPA core guidance – especially differences between countries in the national healthcare sectors, financial opportunities, regional features, and legal basis must be acknowledged. Thus, core guidance can only be an advice with a broad consensus on the main aspects of treatment, but not a complete guideline trying to address all aspects of treatment in each healthcare setting. In summary, this progress report shows the results of a two-step Delphi process regarding the voting of predefined recommendations across Europe. This progress report lays the foundation for a pan-European core and living guidance for the management of schizophrenia, but also points out that for such a process in future a further development of the rules and regulations of how to develop such a guidance is necessary.
